# Measuring the performance of Antarctic Treaty decision‐making

**DOI:** 10.1111/cobi.14349

**Published:** 2024-08-20

**Authors:** Natasha Blaize Gardiner, Neil Gilbert, Daniela Liggett, Michael Bode

**Affiliations:** ^1^ Gateway Antarctica, Centre for Antarctic Studies and Research, School of Earth & Environment University of Canterbury Christchurch New Zealand; ^2^ Antarctica New Zealand Christchurch New Zealand; ^3^ School of Mathematical Sciences Queensland University of Technology Brisbane Queensland Australia; ^4^ Securing Antarctica's Environmental Future Queensland University of Technology Brisbane Queensland Australia

**Keywords:** conservation, environmental governance regime, environmental pressures, human threats, performance indicators, soft law, amenazas humanas, conservación, derecho indicativo, indicadores de desempeño, presiones ambientales, régimen de gestión ambiental, 环境管理制度, 人为威胁, 环境压力, 成效指标, 软法律, 保护

## Abstract

Agreements reached at the Antarctic Treaty Consultative Meetings (ATCMs) are among the primary means for addressing Antarctic conservation and environmental protection issues. However, according to contemporary scholars, Antarctic Treaty decision‐making is becoming increasingly unresponsive to the rising environmental challenges in the region. We assessed the performance of Antarctic Treaty decision‐making by measuring the rate and diversity of decision‐making over the last 6 decades. To measure the rate, we counted the number of inputs and outputs of ATCMs and calculated the time taken for legally binding outputs to enter into force. To measure diversity, we calculated the range of topics addressed by the inputs and outputs of ATCMs. The average number of agreements reached per ATCM increased from 1961 to 2022. Although the diversity of Antarctic topics discussed at ATCMs remained consistently high, the diversity of topics on which legally binding agreements were adopted declined significantly. Antarctic issues—including those of highest priority—are now almost entirely dealt with through nonbinding, soft‐law agreements. It is plausible that this move away from binding decisions reflects a dynamic governance institution evolving to respond to new pressures. However, we suggest that the change reveals a concerning shift in decision‐making behavior and performance, unique to the treaty's history. Soft law is beneficial in some cases, but its overuse diminishes accountability and transparency, significantly reducing the parties’ abilities to understand and measure their performance, including the outcomes and impacts of decisions. Although the rate and diversity of ATCM inputs and outputs provide only a partial view of decision‐making performance, the exploration of these metrics provides a foundation for asking essential questions about the impacts of Antarctic Treaty governance on the region's environmental protection and conservation.

## Abstract (es)

Medida del desempeño de las decisiones del Tratado Antártico

**Resumen:**

Los acuerdos logrados en las Reuniones Consultivas del Tratado Antártico (RCTA) son uno de los principales medios para abordar las cuestiones de conservación y protección ambiental de la Antártida. Sin embargo, según los académicos contemporáneos, la toma de decisiones del Tratado Antártico es cada vez menos receptiva a los crecientes retos ambientales de la región. Evaluamos el rendimiento de las decisiones del Tratado Antártico con la medida del ritmo y la diversidad de la toma de decisiones en las últimas seis décadas. Para medir el ritmo, contamos el número de entradas y salidas de las RCTA y calculamos el tiempo que tardan en entrar en vigor los resultados con vinculación jurídica. Para medir la diversidad, calculamos la gama de temas abordados por las entradas y salidas de las RCTA. El número medio de acuerdos alcanzados por cada RCTA aumentó de 1961 a 2022. Sin embargo, mientras que la diversidad de temas antárticos debatidos en las RCTA se mantuvo constantemente alta, la diversidad de temas sobre los que se adoptaron acuerdos con vinculación jurídica disminuyó significativamente. Las cuestiones antárticas ‐incluidas las de máxima prioridad‐ se abordan ahora casi en su totalidad mediante acuerdos no vinculantes y de derecho indicativo. Es plausible que este alejamiento de las decisiones vinculantes refleje una institución de gestión dinámica que evoluciona para responder a nuevas presiones. Sin embargo, sugerimos que el cambio revela una modificación preocupante en el comportamiento y los resultados de la toma de decisiones, único en la historia del tratado. El derecho indicativo es benéfico en algunos casos, pero su uso excesivo disminuye la responsabilidad y la transparencia, lo que reduce significativamente la capacidad de las partes para comprender y medir su rendimiento, incluidos los resultados y los impactos de las decisiones. Aunque la tasa y la diversidad de las entradas y salidas de las RCTA sólo proporcionan una visión parcial del rendimiento de la toma de decisiones, la exploración de estas métricas proporciona una base para plantear preguntas esenciales sobre los impactos de la gestión del Tratado Antártico para la protección y conservación ambiental de la región.

## 【摘要】

南极条约协商会议 (Antarctic Treaty Consultative Meeting, ATCM) 所达成的协议是解决南极保护和环境问题的主要手段之一。然而, 有当代学者提出, 《南极条约》的决策越来越无法应对该地区日益严峻的环境挑战。本研究通过分析过去六十年来ATCM决策的速度和多样性, 评估了《南极条约》的决策成效。为衡量速度, 我们统计了ATCM的投入和产出数量, 并计算了具有法律约束力的产出生效所需的时间。为衡量多样性, 我们计算了ATCM投入和产出所涉及的主题范围。结果显示, 从1961年到2022年, 每个ATCM达成协议的平均数量有所增加。然而, 虽然在ATCM中讨论的南极议题的多样性一直很高, 但会议通过的具有法律约束力的协议的议题多样性却显著下降。南极问题——包括那些最优先的问题——现在几乎完全通过不具约束力的软法律协定来处理。这种制定不具法律约束力决策的做法似乎反映出一个动态的管理机构正在不断发展, 以应对新的压力。但我们认为, 这一变化揭示了决策行为和成效方面令人担忧的转变, 这在条约历史上是仅有的案例。软法律在某些情况下是有益的, 但其过度使用会削弱问责制和透明度, 显著降低缔约方了解和衡量其成效的能力, 包括决策的结果和影响。虽然ATCM投入和产出的速度和多样性仅提供了决策成效的部分视角, 但分析这些指标为提出有关《南极条约》管理对该地区环境保护影响的基本问题奠定了基础。**【翻译: 胡怡思; 审校: 聂永刚】**

## INTRODUCTION

Over the past 6 decades, human activities in Antarctica have been managed under various instruments of the Antarctic Treaty System (ATS). At the heart of the ATS is the 1959 Antarctic Treaty, which sets aside the region south of 60° southern latitude for peace, science, and international cooperation. The ATS also includes the 1980 Convention on the Conservation of Antarctic Marine Living Resources (CCAMLR) and the 1991 Protocol on Environmental Protection to the Antarctic Treaty (the Protocol), which together establish the Antarctic's environmental conservation and protection regime.

The instruments of the ATS set high environmental standards for Antarctica and the Southern Ocean (McIvor, 2020; Miller, 2011). In Article 2 of the Protocol, for example, the parties commit themselves to the “comprehensive protection of the Antarctic environment,” and Article 7 introduces an indefinite prohibition on mineral resource activities (other than for scientific purposes). Consequently, Antarctic conservation practices have been pointed to as a gold standard (Haward et al., 2012; Koivurova, 2005; Miller, 2011), and Antarctic and Southern Ocean environments have been described as a relatively undisturbed benchmark against which ecosystems around the globe could be compared (Joyner, 1998). However, it is questionable whether the ATS delivers on this soaring rhetoric today. The biodiversity outlook for Antarctica now reflects the same concerning trends found elsewhere on the planet (Chown et al., 2017). Despite its remote location, the Antarctic faces escalating pressures from human activities (Brooks, 2013; Chown et al., 2022; IPCC, 2019; Jenouvrier et al., 2017; Lee et al., 2022), which put its unique values at risk (Hughes et al., 2018). The ATS has been criticized for failing to adopt substantive instruments to address ongoing challenges, such as for tourism (Bastmeijer et al., 2023) and bioprospecting activities (Leary & Walton, 2010).

The ATS is experiencing increasing internal and external pressures. As a regime, the ATS does not exist in isolation but is nested within a broader regime complex and within an overarching global political order (McGee & Haward, 2019; Scott, 2019; Young, 2011, 2023). Although the ATS has been lauded for its ability to make progress even during periods of external conflicts among its members (Joyner, 1984; Haward, 2019; Lord, 2020), recent events suggest that this resilience may be in decline. For the first time, the Antarctic Treaty Consultative Meeting (ATCM) in 2022 was unable to reach a consensus on the final report of the meeting due to disagreements regarding statements made on Russia's invasion of Ukraine (ATCM, 2022).

The timeliness of Antarctic decision‐making has also come under scrutiny, with scholars concluding that the speed of policy development—particularly on high‐priority Antarctic issues—is too slow (Anne et al., 2018; Bastmeijer et al., 2023; Chown et al., 2012; Convey et al., 2012; Green, 2022; Hemmings, 2018; Hughes & Pertierra, 2016; Liggett et al., 2017; Phillips et al., 2022). These critiques assert that the rate of Antarctic decision‐making is inadequate, but empirical evidence to support these claims is lacking. In particular, the rate at which the ATCMs consider inputs and create outputs—important precursors to real‐world change—has not been measured.

We quantitatively measured decision‐making by the ATCMs over the last 6 decades. We measured the rate of Antarctic Treaty decision‐making by counting the number of meeting inputs and outputs produced each year. We also measured the diversity of Antarctic Treaty decisions by calculating the range of topics addressed by the inputs and outputs of the ATCMs. Measuring the performance of international regimes is challenging (Young, 2011), and these analyses were not meant to provide a complete assessment of Antarctic governance. In particular, the inputs and outputs of a decision‐making process should not be confused with its outcomes or impacts. An effective governance system in the Antarctic will need to consider and address a wide range of rapidly emerging challenges; therefore, measuring the rate and diversity of ATCM decision‐making is an important foundation for asking more profound questions about the overall performance of Antarctic governance.

To help frame our analyses, we began with a brief overview of Antarctic Treaty decision‐making processes and then considered our findings in the context of exogenous and endogenous factors that influence decision‐making. Finally, we raise several questions that build on our analyses that are pertinent for researchers or practitioners interested in the future of Antarctic governance.

### Antarctic Treaty decision‐making

**FIGURE 1:**
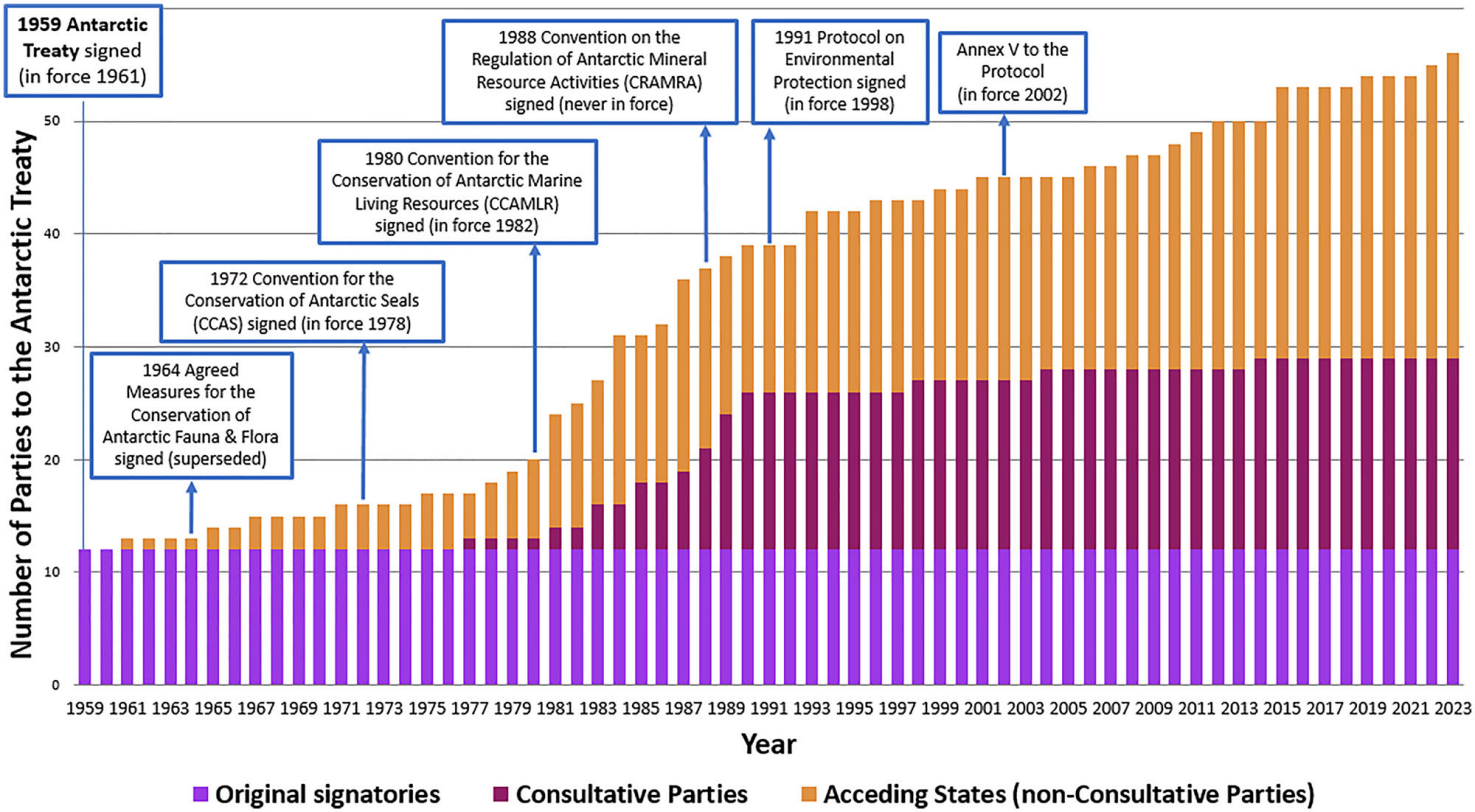
The number and type of signatories to the Antarctic Treaty 1959–2023 (includes the substantive legally binding instruments that comprise the ATS).

The Antarctic Treaty establishes the ATCM as the central forum for Antarctic decision‐making (Article IX). To varying degrees, all national delegations, observers, and experts who attend the ATCMs can inform the decision‐making process by providing formal inputs (meeting papers) that address the meeting agenda items (Joyner, 1998). At the time of writing, there were 56 signatories to the Antarctic Treaty (Figure 1). Of those, 29 states—referred to as Antarctic Treaty Consultative Parties (ATCPs)—have decision‐making rights. The ATCPs include the 12 original signatories to the Treaty and those states that have since demonstrated their interests in Antarctica through substantial scientific activities on the continent (Article IX [2]). The other 27 Antarctic Treaty Non‐Consultative Parties that attend ATCMs can submit certain types of inputs and contribute to discussions but do not have decision‐making rights.

**FIGURE 2:**
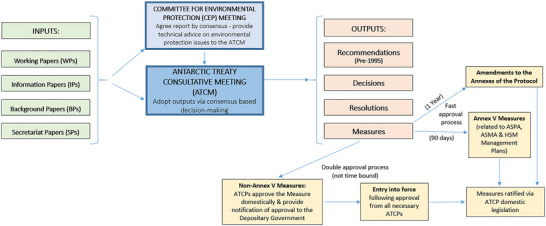
Simplified decision‐making processes of the Antarctic Treaty Consultative Meeting (ATCM), identifying meeting inputs, outputs, and the approval mechanisms for the ratification of legally binding measures.

A simplified schematic of the formal ATCM decision‐making process is in Figure 2. The ATCM is the decision‐making body, and the Committee for Environmental Protection (CEP) is the technical advisory body to the ATCM (established in 1998), tasked with the provision of recommendations and advice to the ATCM relating to the implementation of the Protocol. Decision‐making inputs in the form of meeting papers are submitted to each of these bodies for discussion, but it is in the ATCM that ATCPs adopt agreements by consensus, which is reached in the absence of objection. There is no formal voting process. Consensus may result in several types of outputs that are either legally binding or nonbinding. Nonbinding outputs include resolutions and decisions. Binding outputs, called measures, enter into effect through a double approval process (Figure 2). First, ATCPs agree to adopt the measure by consensus at an ATCM. Second, ATCPs must give effect to the measure in their national legal and administrative systems and then notify the depositary government (the United States) and the Antarctic Treaty Secretariat. Once all ATCPs that were entitled to be present at the time of adoption have undertaken both steps, the measure enters into effect. An exception to this double approval process are measures adopted in relation to Annex V (area protection and management) of the Protocol (entry into force 2002). These automatically enter into effect 90 days after their adoption. Although measures are binding on the nationals of signatory parties, they are enforced at the domestic level by relevant competent authorities; the Antarctic Treaty has no enforcement mechanisms.

A much broader range of actions contribute to the Antarctic Treaty decision‐making process (e.g., informal bilateral discussions, intersessional work) than those depicted in Figure 2. However, our analyses focused on the formal inputs and outputs of the decision‐making process of the ATCMs.

## METHODS

We viewed Antarctic governance through a constructivist lens, which emphasizes the roles of shared norms and practices in shaping state behavior toward shared international challenges (Vogler, 2022), including environmental stewardship (Young, 2011). Although power and state interests play a central role in Antarctic affairs, we focused our evaluation of the Antarctic environmental regime on observing the operation of its collective problem‐solving mechanisms, specifically the ATCMs. In doing so, we measured 5 indicators of ATCM decision‐making performance from 1961 to 2022.

### Rate of submission of meeting inputs (indicator 1)

There are currently 4 types of formal inputs to the ATCMs. We focused on working papers (WPs) and information papers (IPs). WPs can be submitted by ATCPs and observers, relate to specific agenda items, and must be discussed on the meeting floor. The ATCPs may make decisions on the basis of the recommendations included in WPs. IPs can be submitted by Antarctic Treaty Consultative and Non‐Consultative Parties, observers, and invited expert organizations and may provide supplementary information in relation to a WP or information of relevance to a specific agenda item. They may be discussed on the meeting floor.

Background papers (BPs) and secretariat papers (SPs) are also submitted to the meetings, but BPs are not discussed, and neither BPs nor SPs require decisive action. We therefore focused on WPs and IPs because these documents are a primary driver of meeting discussions and provide the main impetus for the meeting dialogue from which formal decisions may arise (Sampaio, 2022). However, there is not a direct relationship between these meeting inputs (WPs and IPs) and the meeting outputs. The ATCPs can make decisions that are not based on the information or recommendations provided by WPs. Furthermore, not all WPs call for the adoption of a formal output. A WP can include soft recommendations, such as the need for continued discussion on an issue. Accordingly, the relationship between paper submissions and meeting decisions is not linear. A decision may arise from one meeting paper at the same meeting, or it may take multiple meeting paper submissions from multiple countries over several meetings before a formal decision emerges (here we are referring to decisions made by the parties, not the meeting outputs known as ‘decisions’). Nonetheless, the extent and diversity of these meeting inputs do have an influence on the nature and extent of the meeting outputs, which is why we included them in our analyses.

### Rate of adoption of meeting outputs (indicator 2)

Our second metric of decision‐making rate was the number of regulatory mechanisms agreed at each of the ATCMs. From the Antarctic Treaty's inception until 1995, ATCM outputs were adopted as recommendations (Figure 2), all of which needed double approval (Sampaio, 2022). However, issues soon arose regarding the legal status of those recommendations that had not yet been ratified by all relevant ATCPs (Joyner, 1998). In a move to improve the efficiency and clarity of the decision‐making process, in 1995, the ATCPs separated outputs into 3 categories (measures, resolutions, and decisions) (Decision 1, 1995 [ATCM, 1995]): measures are legally binding agreements; resolutions are hortatory agreements (nonbinding soft law); and decisions are agreements that specifically relate to organizational and administrative matters (nonbinding soft law).

Our analyses included all recommendations prior to 1995 and all measures, decisions, and resolutions since then up to 2022. In total, 725 outputs have been adopted over the 44 ATCMs across the study period. Of those, 204 were recommendations, 240 were measures, 118 were decisions, and 163 were resolutions (Antarctic Treaty Secretariat, 2024). The ATCPs can make decisions at the ATCMs that do not result in the formal outputs as outlined above. These are largely administrative agreements reached during meeting discussions and can be found in the meeting reports. They appear as *other* in the ATS database (Meeting Documents Archive [https://www.ats.aq/devAS/Meetings/DocDatabase?lang=e]). There are only 28 of these across the study period. For the purposes of our analyses, which focused on agreements reached with respect to Antarctic Treaty Article IX, they were excluded.

### Rate of entry into force of legally binding outputs (indicator 3)

We determined the amount of time it took for each binding agreement to enter into force once it was adopted by an ATCM. That is, how long in years it took for each binding agreement to enter into force from 1961 to 2022.

### Diversity of topics addressed by inputs and outputs (indicators 4 & 5)

The number of agreements reached (outputs) by each ATCM is an important metric of system performance but does not indicate whether the ATS is addressing a wide range of policy challenges. We therefore also examined the diversity of decision‐making as another metric of performance. We identified 2 indicators for diversity: diversity of Antarctic issues submitted to the ATCM for discussion (i.e., through WP inputs) and diversity of issues being addressed through the adoption of outputs.

The inputs and outputs of the ATCMs address a wide range of Antarctic topics. The Antarctic Treaty Secretariat Database has categorized each of the inputs and outputs into one of 44 broad categories (at the time of writing), which range from bioprospecting, to marine living resources, to waste management and disposal (and so forth). Given that WPs are the key inputs driving ATCM discussions, we considered the topics addressed by WPs to represent the range of Antarctic challenges that decision makers consider a high priority at any given time. Likewise, we considered the outputs to reflect the ability of the ATCPs to reach a consensus on how these challenges should be addressed through international law, noting that some WPs do not call for the adoption of a formal output.

For categorical data, such as the topics of inputs and outputs, which we combined under the umbrella term *documents*, many different methods were available to measure diversity. We applied Simpson's diversity index (ϕ):

(1) $$\phi =1-\left(\frac{\sum_{i}n_{i}\left(n_{i}-1\right)}{N\left(N-1\right)}\right),$$

where $n_{i}$ is the number of documents that belonged to category i and N is the total number of documents. We interpreted ϕ as the probability that 2 randomly chosen documents would belong to different categories. When all documents belonged to the same category (i.e., $n_{1}=N$), then ϕ was equal to 0, reflecting a total lack of diversity. When the number of documents in each category was equal, ϕ began to approach 1, which was the maximum amount of diversity. For example, if 440 documents were submitted to the ATCMs, and 100 of them were in each of the ATS Database's 44 categories, then ϕ≈0.98.

### Methodological limitations

The original data we used were available at the Antarctic Treaty Secretariat meeting documents archive (https://www.ats.aq/devAS/Meetings/DocDatabase?lang=e). The Antarctic Treaty Secretariat database is the central depositary for Antarctic Treaty policy documents. It was established in 2003 and contains the documents of the ATCM. The ATS database contains a large number of meeting documents, including 1749 WPs and 3086 IPs over our study period. The Antarctic Secretariat has enormously improved the storage and availability of ATS data, but several key limitations remain. First, the lack of a permanent secretariat means that prior to 2004, each ATCP hosting the ATCM was responsible for the compilation of meeting documents, which resulted in the legislative record having discontinuities, gaps, and duplications. Our analyses would mirror any such gaps. Second, prior to 2004, ATCPs also inconsistently reported on the process of superseding or replacing prior measures. Once the permanent secretariat was established, they declared a number of prior recommendations as “spent” (i.e., 24 recommendations at the 25th ATCM and 13 recommendations at the 30th ATCM) (Figure 5).

The database includes documents that relate to 3 types of meetings among the Antarctic Treaty Parties: ATCMs, SATCMs (Special Antarctic Treaty Consultative Meetings), and ATMEs (Antarctic Treaty Meeting of Experts). We omitted the small number of agreements made at SATCMs (none are made at ATMEs) because they are not the main avenue for formal decision‐making. Binding agreements adopted at SATCMs include the major agreements (CCAMLR, Convention on the Regulation of Antarctic Mineral Resource Activities [CRAMRA], and the Protocol) and a small number of agreements related to consultative party applications. These agreements significantly contribute to regime building but are not directly comparable to more routine decisions made at ATCMs, which are our focus. Including them in our computational analyses would either inappropriately have treated them as all other measures taken at ATCMs or we would have needed to weight them differently, which would have distorted the results for years in which these SATCM agreements had been adopted. This generic exclusion of SATCMs meant that 2 measures, 1 decision, and 2 resolutions adopted at SATCM‐XII (The Hague) were also excluded.

## RESULTS

### Rate of submission of meeting inputs and outputs

**FIGURE 3:**
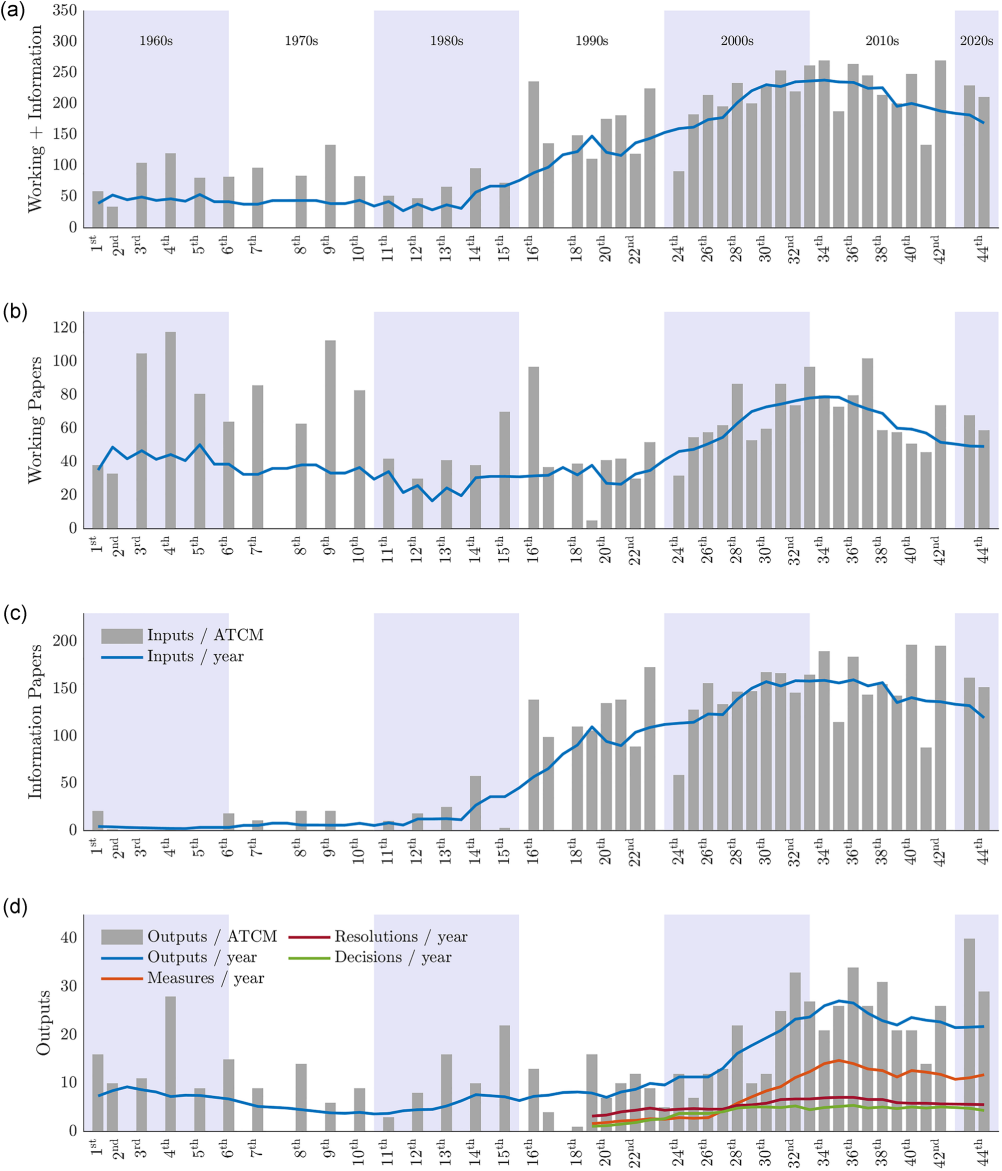
The Antarctic Treaty Consultative Meeting (ATCM) inputs and outputs 1961–2022 (dark gray bars, number of inputs or outputs per ATCM; colored lines, moving averages of inputs or outputs per year; shading, decades): (a) pooled number of working papers (WPs) and information papers (IPs), (b) WP submissions, (c) IP submissions, and (d) outputs adopted (blue line, moving average for all outputs adopted from 1961 to 2022—recommendations [1961–1994], measures, resolutions, decisions [1995–2022]; orange, red, and green lines, moving averages for the different types of outputs adopted per year from 1995 until 2022 (measures, resolutions, and decisions, respectively). There was no ATCM in 2020, and ATCMs prior to 1991 occurred biennially. The years without ATCMs were not used in the calculations of the moving averages.

The number of WP and IP submissions per ATCM was relatively stable from 1961 to 1980; the rate of IP submissions was particularly low (Figure 3a–c). The number of IPs per meeting increased from the mid‐1980s, and the WPs began to increase around 2000. The rate of submission of both inputs stabilized at the start of the 2010s. The IPs showed the most rapid increase overall, becoming the dominant type of submission, at an average of 150 submissions per year.

The acceleration of inputs beginning in the mid‐1980s occurred around the same time that membership to the Antarctic Treaty was increasing (Figure 1). Resource issues were foremost during this period, with the CCAMLR taking effect in 1982 and negotiations underway toward an Antarctic minerals regime (Hemmings, 2014; Sampaio, 2022). Several developing nations contributed to the membership expansion by rapidly acceding to the Antarctic Treaty to participate in the minerals debate and potentially gain privileged access to Antarctic resources (Arpi & McGee, 2022; Haward & Jackson, 2023). The ATCPs eventually signed the minerals convention (CRAMRA) in 1988, but the refusals of France and Australia to ratify it meant that it never entered into force (Haward & Jackson, 2023). In a flurry to rebuild consensus among the ATCPs and regain the trust of the international community (among other motivating factors), the Protocol was signed in 1991 (in force in 1998). The Protocol indefinitely bans Antarctic mineral resource activities (other than for scientific purposes) through Article 7 (Dodds, 2010). These significant topics and the inclusion of more ATCPs providing inputs on more issues likely contributed to the increase in paper submissions in the 1990s and 2000s.

The average number of outputs agreed per year showed a period of relative stasis from 1960 to the 1990s, followed by an acceleration lasting from the 1990s to the start of the 2010s (Figure 3d). The years since have fluctuated, a broad trend that mirrored those found for the inputs (Figure 3a–c). When we separated these measures into their different categories following 1995, it became apparent that the legally binding measures were largely responsible for changes in the overall outputs, whereas the nonbinding resolutions and decisions remained relatively consistent across this period.

Figure 3 paints an optimistic picture of responsive and active Antarctic governance. Over time, the ATCPs were discussing and dealing with an increasing number of Antarctic issues at ATCMs. Moreover, they were translating this increased workload into outputs—particularly legally binding outputs—in response. A range of drivers may underpin these trends. For example, the establishment of the CEP in 1998 meant that there was a second body operating alongside the ATCM with a mandate to increase attention on environmental protection issues. It is plausible that this led to an increase in inputs on issues of environmental concern and consequently an increase in outputs addressing those.

**FIGURE 4:**
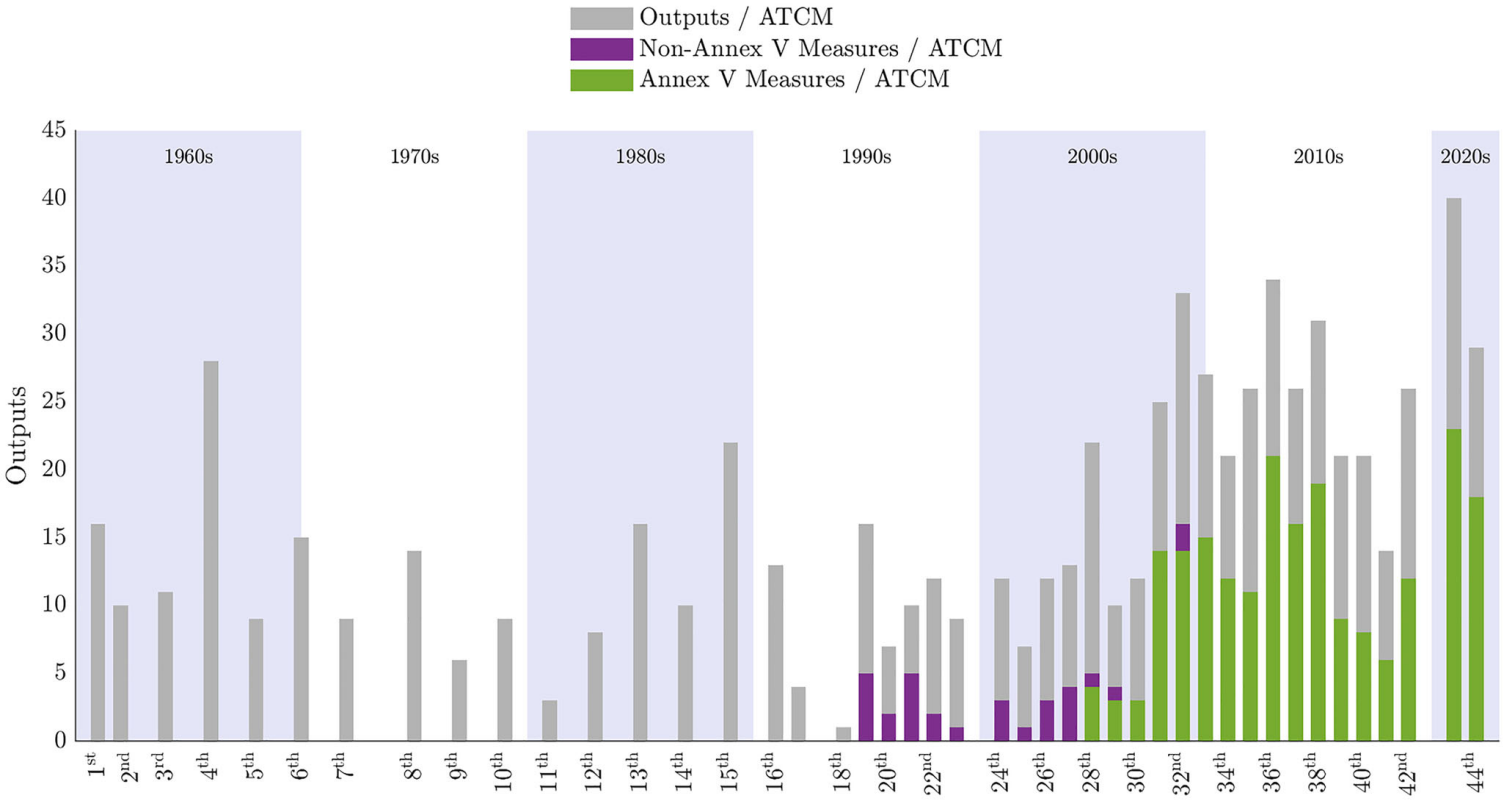
The Antarctic Treaty Consultative Meeting (ATCM) outputs 1961–2022 (light gray bars, counts of all outputs adopted per ATCM; purple bars, counts of all legally binding measures adopted per ATCM that do not relate to the Protocol on Environmental Protection Annex V; green bars, counts of all legally binding measures adopted per ATCM that relate to Annex V; shading, decades).

However, the next step in our analyses (Figure 4) highlighted an important caveat to this optimistic picture by distinguishing between the types of outputs being produced. In particular, it showed which of the legally binding measures adopted after 2002 were passed under Annex V to the Protocol, which provides for the designation of Antarctic specially protected areas (ASPAs), Antarctic specially managed areas (ASMAs), and the listing of historic sites or monuments (HSMs). We found that the post‐2002 acceleration of outputs apparent in Figure 3d was largely attributable to Annex V measures, whereas the number of non‐Annex V measures declined steadily after 1995. In fact, since 2009, the only binding agreements adopted were related to ASPA and ASMA management plans or HSMs. No other issues seemed to require attention by means of legally binding outputs.

From 2002 to 2007, a single measure was used at each meeting to adopt multiple new or revised ASPA or ASMA management plans. For example, measure 1 (2002) adopted 13 revised ASPA management plans. In 2008, changes were made to the decision‐making process so that each new or revised ASPA or ASMA management plan was adopted by a separate measure. For example, in 2008, 14 separate measures (1–14) were adopted to provide for the designation of 4 new ASPAs and the approval of 10 revised ASPA management plans. These changes inflated the number of Annex V measures per ATCM after 2008.

### Rate of entry into force of legally binding outputs

**FIGURE 5:**
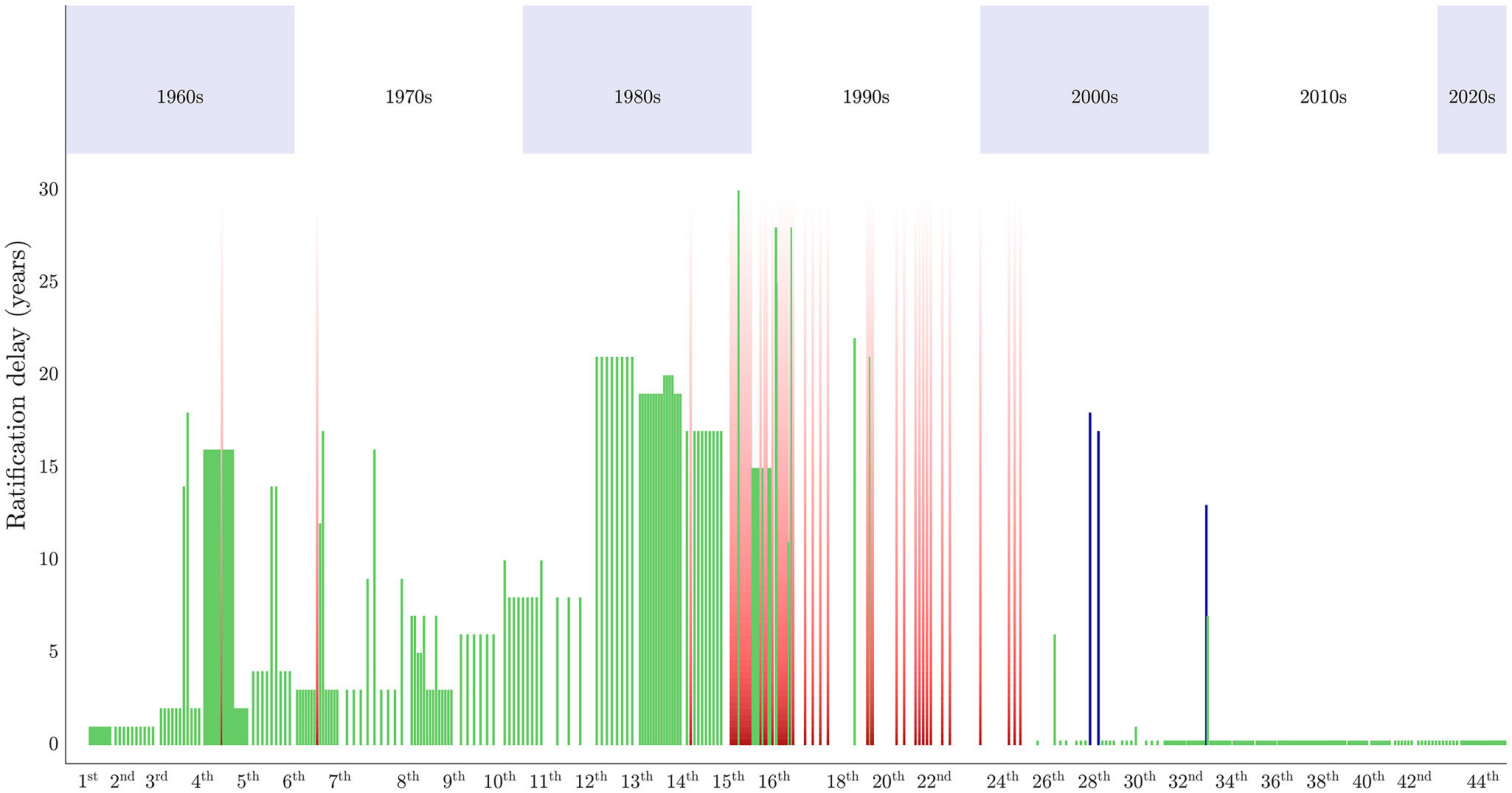
Delay between year of the Antarctic Treaty Consultative Meeting (ATCM) adoption of binding measures and year of entry into force (each line, one measure passed at the ATCM identified on the *x*‐axis; green lines, measures that entered into force [length represents length of time it took for this to occur]; red line fading, measures adopted that never entered into force due to being superseded by a more recent measure or because they became obsolete with no further action required from the parties; blue lines, measures adopted by the ATCM not yet in effect; shading, decades).

In terms of ratification delay, we identified 3 phases across the history of the ATCMs (Figure 5). Parties steadily adopted outputs throughout the 1960s, 1970s, and 1980s (Figure 3d). In this first phase, there was a gradual increase in the time it took for these outputs to enter into force following ATCM adoption. By the 12th ATCM in 1983, some recommendations were taking up to 21 years to enter into force. The longest ratification delay was for Recommendation XV‐5 (1989) on Environmental Monitoring Activities, adopted at the 15th ATCM. Recommendation XV‐5 finally entered into force at the 42nd ATCM in 2019, some 30 years later.

In the late 1980s and the 1990s, there was a discrete change in the ratification dynamics. In this second phase, we began to see the adoption of measures at ATCMs that never entered into force (red lines in Figure 5). Many of the measures adopted throughout this period related to area protection and management and were eventually superseded by Annex V measures in 2002.

Finally, in the third phase after 2000, delays between ATCM approval and ratification decreased abruptly, becoming almost uniformly immediate. Although this result suggested an accelerating governance process, closer inspection qualified this optimism. All of these immediately ratified measures were under Annex V. These have a tacit approval mechanism, whereby they automatically enter into force 90 days following their adoption (Figure 2), significantly reducing the problematic ratification delays of the earlier decades. However, this meant that the apparent acceleration of the ratification process post‐2000 was actually a result of the ATCM measures dealing primarily with area protection and management.

The exceptions in this third phase were a set of measures (dark blue lines in Figure 5) that did not relate to area protection and management and that are yet to enter into force. That is, they have been adopted at an ATCM but have not yet been ratified. Since the 1995 recategorization of policy outputs, only 6 measures have been adopted that do not relate to area protection and management, and of those, 3 are not yet in force. These include Measure 4 (2004) on Tourism and Non‐Governmental Activities, Measure 1 (2005) on Annex VI (Liability), and Measure 15 (2009) on Landing of Persons from Passenger Vessels.

### Diversity of topics addressed by inputs and outputs

**FIGURE 6:**
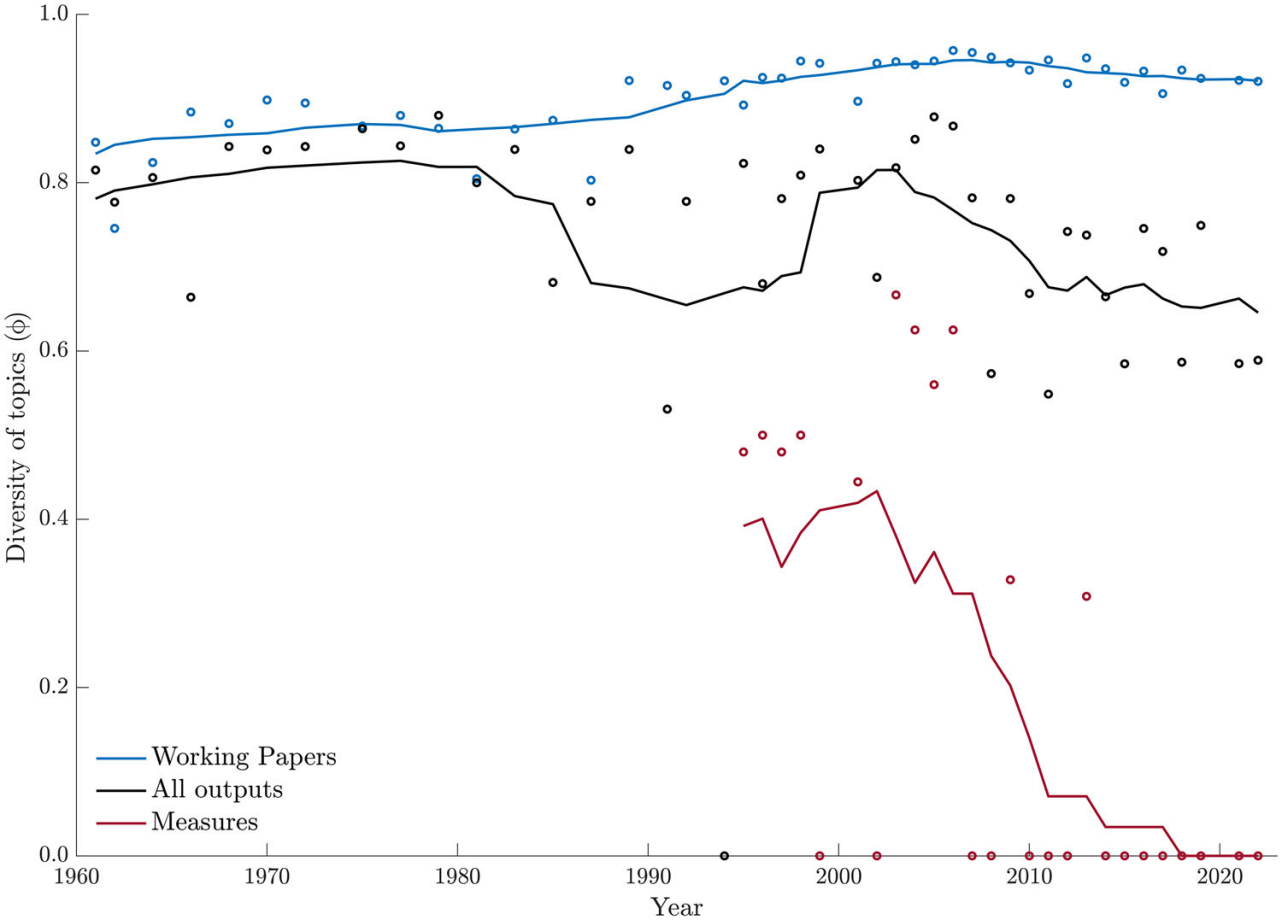
Diversity of Antarctic Treaty Consultative Meeting (ATCM) inputs and outputs 1961–2022 (blue, inputs [working papers only]; black, all binding and nonbinding outputs [recommendations, measures, resolutions, decisions]; red, binding outputs after 1995 [measures only]; circles, values for each ATCM; lines, moving average with a 10‐year window).

WPs submitted to the ATCMs over the last 6 decades encompassed an impressively high diversity of topics (Figure 6, blue line). That is, the ATCPs discussed a very broad range of Antarctic issues at each ATCM since 1961. With respect to outputs, during the 1960s and 1970s, the diversity of issues covered by the agreements reached by the ATCPs was almost consistent with the range of issues discussed at the meetings (Figure 6, black line). By contrast, the 1980s and early 1990s saw a decline in the average diversity of issues among adopted outputs. A factor in this observed decline in the 1980s was attributable to the 18th ATCM in 1994, which adopted just one recommendation on tourism, resulting in zero diversity for that year.

Overall output diversity remained high through the mid‐1990s and 2000s (matching earlier levels of diversity) before declining again from 2002 until 2022. Over the last 2 decades (approximately), the diversity of issues being discussed at the ATCMs has been higher than ever before, but the diversity among outputs showed a decline. Nevertheless, diversity of overall outputs remained relatively high across the history of the ATCMs.

However, this consistent overall diversity masked a bifurcation between the diversity of the legally binding measures and the diversity of the nonbinding decisions and resolutions. The diversity of issues addressed by the measures fell rapidly after 1990, toward complete homogenization (Figure 6, red line). There has been a total lack of binding agreements on issues not related to area protection and management since 2009 and only a few measures on other topics from 1995 to 2009. By contrast, the ATCPs continued to adopt hortatory resolutions and decisions on a great diversity of issues.

## DISCUSSION

Decision‐making performance by any international regime is important (Sommerer et al., 2022). It reflects the effectiveness and productivity of the regime objectives (Young, 1999) and its ability to “contribute […] significantly to the solution of the major problems addressed by it” (Stokke & Ostreng, 1996, p. 113). Decision‐making performance also provides an indicator of a regime's ability to evolve and meet contemporary challenges (Alter & Raustiala, 2018; Sommerer et al., 2022).

There is a clear expectation in the Antarctic Treaty that the adoption and ratification of new agreements will be a primary output of the decision‐making processes of regular Antarctic governance meetings. Article IX (1) of the treaty sets out 3 purposes for holding ATCMs: exchanging information, consulting on matters of common interest, and formulating, considering, and recommending to their governments, measures in furtherance of the principles and objectives of the treaty. The ATCM Recommendation III–VII (1964) encourages new ATCPs to ratify all previously agreed binding measures and reiterates that measures are important by emphasizing that they contribute to the overall functioning and implementation of the system. The parties have therefore repeatedly emphasized the critical need to adopt and ratify binding outputs.

The ATCPs have made 2 significant changes to the decision‐making process aimed at improving its efficiency. First, in 1995, they separated recommendations into measures, resolutions, and decisions to clarify those agreements that were intended to be legally binding. Second, in 2002, the tacit (or fast approval) mechanism for measures adopted in relation to the Protocol's Annex V became effective (ASPA, ASMA management plans, and HSM listings). The first of these certainly clarified the distinction between those agreements intended to be mandatory and those of a hortatory nature, and the second has increased the rate at which certain (Annex V related) binding agreements become effective (Figure 5). But have these changes had unintended consequences?

Our analyses showed that since the mid‐1990s, the nature of Antarctic Treaty decision‐making has changed. Despite the self‐proclaimed importance of measures, the ATCPs have revealed a recent preference for adopting hortatory resolutions over legally binding measures. Paradoxically, although the diversity of issues being addressed by the measures has rapidly declined, the diversity of Antarctic issues that are of concern to the parties has remained high. The majority of challenges facing the Antarctic today are therefore being dealt with through regulations that rely entirely on the voluntary action of the parties. Furthermore, between 1995 and 2022, only 6 of 240 measures have dealt with matters not related to area protection and management; of those, 3 are not yet in effect. Of the 234 Annex V measures entered into force during that period, 214 were routine updates to ASPA management plans; 20 adopted new ASPA management plans. Moreover, although it may seem like the parties are spending ample time agreeing on measures on area protection and management, researchers are becoming increasingly critical of the protected area system for being unrepresentative of the continent's biodiversity. A more systematic conservation planning approach is deemed more effective and readily achievable but is currently out of sight (Burrows et al., 2023; Coetzee et al., 2017; Shaw et al., 2014).

By contrast, Antarctic tourism provides an example of an important issue that has resulted in predominantly soft‐law regulations (largely implemented by the tourism industry). Tourism features as a top‐tier priority in the work plans of the ATCM and CEP, and the increasing number of tourists has been identified as a threat to Antarctic biodiversity (Lee et al., 2022), although the cumulative environmental impacts of tourism activities remain poorly understood (Tejedo et al., 2022). For these reasons, researchers have increasingly urged Antarctic decision makers to develop and implement a comprehensive and binding policy framework for tourism (Enzenbacher, 1992; Lamers et al., 2012; Liggett & Stewart, 2017; Verbitsky, 2013), but these calls to action have resulted in little binding regulatory change (Cajiao et al., 2021; Makanse, 2024). Since 1995, over 30 hortatory resolutions have been adopted on a variety of tourism‐related matters alongside only 2 binding measures, neither of which are yet in force (Bastmeijer et al., 2023). The resolutions include the adoption of several nonmandatory visitor site guidelines, which provide site‐specific information and guidance for the appropriate management of Antarctic tourism activities ashore. However, adherence to these guidelines relies largely on the cooperation of the tour operators, with any governmental enforcement left to each party individually to decide.

On the one hand, a preference for soft law does not necessarily suggest a decline in decision‐making performance. According to Joyner (1998), “soft law allows for greater flexibility [and] governments are often more willing to be innovative when the product is not explicitly binding” (p. 414). Moreover, soft law can lay the foundations for negotiating legally binding laws later (Abbott & Snidal, 2000), which was the case during the lead up to the legal instruments agreed in the early decades of the ATS (Joyner, 1998). Other benefits of soft law in international legal settings include, inter alia, easier implementation, a reduction of costs associated with harder legislation, more effective ways to deal with uncertainty, crystallizing a trend toward new norms, and increased compromise and cooperation (Abbott & Snidal, 2000; Sampaio, 2022; Shelton, 2011). The latter, of course, requires a convergence of interests and values among those who are expected to implement the soft law.

On the other hand, binding decisions, although often more difficult to achieve, provide clearer mandatory instructions for domestic implementation, incentivize a consistency of standards among ATCPs, and can be used to hold states to account for their actions (or inactions) (Joyner, 1998)—although actual enforcement is rare. The double approval process for (non‐Annex V) measures also provides a feedback mechanism whereby ATCPs must provide confirmation of having domestically ratified these policies. Hortatory agreements are only likely to be implemented domestically if they are not too costly for governments in fiscal, political, or reputational terms (Sampaio, 2022). In contrast to the process required for measures, there is no formal requirement for the ATCPs to report on the implementation of resolutions following their adoption. This lack of accountability makes it difficult to understand to what extent parties have acted upon these agreements, if at all. Therefore, the observed preference for soft‐law agreements has resulted in a loss of accountability and transparency in Antarctic governance—both of which are fundamental principles for the maintenance of an institution's legitimacy and strength (Buchanan & Keohane, 2006; Yermakova, 2021).

Among a range of factors that influence Antarctic Treaty decision‐making practices, we point to 3 overarching drivers that we see as particularly relevant in the context of our results. First, the growth and diversification of Antarctic Treaty membership have inevitably changed the governance dynamics over time, with ATCPs having to navigate an increasingly diverse array of values, interests, and domestic legislative arrangements (Bastmeijer & Lamers, 2013; Engelbertz et al., 2013; Ferrada, 2018; Flamm, 2022; Gardiner et al., 2023; Hemmings, 2018; Lord, 2020; McGee et al., 2020; Sampaio, 2022). Second, institutional constraints that impede decision‐making efficiency are embedded in the structures and processes of the ATS itself. Notably, reaching agreement has become increasingly difficult under the consensus‐based decision‐making model on which the ATS is founded (Bastmeijer et al., 2023; Brooks, 2013; Chaturvedi, 2019; Gardiner, 2020; Goldsworthy, 2022; Yermakova, 2021). Third, the challenges faced by ATCPs are diverse and complex and demand the right expertise, political will, and determination to achieve tangible outcomes. Witness, for example, the technical complexity, practical implications, and lengthy timetables involved in dealing with the Liability Annex to the Protocol (Hemmings, 2018; Huber, 2011; Hughes & Convey, 2014; Sampaio, 2022), the regulation of Antarctic biological prospecting (Herber, 2006; Jabour‐Green & Nicol, 2003; Joyner, 2012; Leary & Walton, 2010), or the regulation of Antarctic tourism activities (Bastmeijer et al., 2023; Cajiao et al., 2021; Dodds, 2010; Liggett & Stewart, 2020; Liggett et al., 2011; Tejedo et al., 2022).

Like the global order, Antarctic governance is a complex system in which tipping points may lead to critical transitions or nonlinear changes. An historical example of such a transition was the speedy turnaround of an Antarctic mining convention to a comprehensive environmental framework reserving Antarctica as a “natural reserve, devoted to peace and science” (Protocol Article 2). It is plausible that the ATS may undergo similar critical transitions in the future, in a continued pattern of punctuated equilibrium (Young, 2010). In the context of our findings, this means that it is not reliably possible to forecast the performance of Antarctic Treaty decision‐making beyond the present moment.

It is also conceivable that our findings reflect a natural evolution of the ATS moving through a series of institutional phases (Sampaio, 2022). However, there appears to be no clear evidence that the recent decline in binding measures is the result of a collectively agreed strategy by the ATCPs. Nor is there evidence of a shared understanding among Antarctic decision makers that the current system is adequate, with no further strengthening required through the adoption of binding measures. Indeed, the recent Decision 6 (2023) to begin a process to develop a more comprehensive policy framework for Antarctic tourism management is a demonstration of the expressed need for further legal innovation. Instead, the observed trends away from binding agreements appear to reflect a path of least resistance for the ATCPs in a progressively challenging geopolitical climate. The institutional changes to the decision‐making process made in 1995 and the separation of binding and nonbinding meeting agreements enabled the preference for soft‐law policy‐making. This legal softening would potentially reflect a strengthening of the system if it were caused by a convergence of values and interests among the parties; that is, if the parties were increasingly able to develop shared sets of norms and values that could be enshrined in soft‐law agreements because there was a collective motivation to push in the same direction. However, as we have outlined, it appears as though there is an increasing divergence of views among (at least some of) the parties. We therefore view the shift away from binding agreements as symptomatic of a decline in the decision‐making performance of the parties over time, which is at odds with the obligations laid out in the treaty's Article IX (1) and appears increasingly unresponsive to the growing pressures on the Antarctic environment (Bloom, 2022; Brooks, 2013; Chown et al., 2017, 2012; Convey et al., 2012; Ferrada, 2018; Leihy et al., 2020; Rintoul et al., 2018).

Options to improve the efficiency of ATCM decision‐making have been considered in the past. In 2002, the ATCM considered a proposal by the United Kingdom (2002) to introduce a tacit approval process for all measures in an attempt to accelerate their entry into force. The United Kingdom noted the examples of tacit approval processes that already exist, including the various amendment provisions contained in each of the Protocol's Annexes and in Article 6 of Annex V to the Protocol, which provides the fast‐track approval mechanism for the adoption of protected and managed area management plans. At that time, a consensus on the proposal was not reached.

Among many potential opportunities for improvement, consideration could also, for example, be given to improving the visibility of the domestic response action taken by the parties with respect to all decisions reached at each ATCM, for resolutions as well as measures. Not all resolutions require parties to take action in their domestic systems, but for some, it is implied. It would be useful to know, for instance, the extent to which and how adherence to site‐specific guidelines for visitors is being encouraged or how the guidance material for minimizing the risks of introducing non‐native species is being applied. A feedback process that is otherwise absent for decisions made through resolutions would be consistent with the information exchange provisions of Article IX of the Antarctic Treaty, as well as those of Article 17 of the Protocol.

Our results highlight a concerning shift in the nature and performance of Antarctic Treaty decision‐making. However, we have only sought to quantify inputs and outputs as a measure for decision‐making performance, which tells us little about what the observed trends mean at the level of governance outcomes and impacts. For instance, what does an increasing preference for soft law mean for the ATCP's ongoing compliance with the rules and norms of the regime, or the ability of the ATS to handle emerging needs for governance? Given the lack of effective enforcement or compliance mechanisms available to the parties, is there really a real‐world distinction between soft and hard law? Furthermore, what do the observed trends mean in the context of different Antarctic governance issues, given that soft law may be of greater or lesser value depending on the problem being addressed? On a broader scale, how does Antarctic Treaty decision‐making compare with other international environmental governance arenas elsewhere? Young (2011) suggests that there must be a shift in attention from the intensive study of individual regimes to more encompassing analyses that consider institutional interplay across regime complexes (Keohane & Victor, 2011), a line of inquiry that deserves more attention in the Antarctic.

With regard to the importance of interactions between and within regimes, Antarctic Treaty decision‐making and Antarctic governance more broadly do not operate in a vacuum (e.g., see Bastmeijer et al., 2023; Ferrada, 2018; Hemmings, 2017; Joyner, 2011; Liggett et al., 2017; McGee & Liu, 2019; Sampaio, 2022; Yermakova, 2021). The ATS is nested in regional and global regime complexes, which are similarly nested in the prevailing global order (McGee & Haward, 2019; Young, 2023). The global order is currently experiencing shifts in the distribution of power among its leading members and is witnessing a rise in the influence of nonstate actors (e.g., private companies) (Young, 2023). The extent to which these global conditions of uncertainty have influenced or are influencing the performance and decision‐making of the ATCPs is beyond the scope of this paper but merits further attention (Press & Bergin, 2022).

Perhaps of even greater significance, if one characterizes the ATS as a complex regime, efforts should be made to understand the systemic forces, constitutive pressures, and political tipping elements that may give rise to critical transitions in Antarctic governance in the future (Young, 2023). Importantly, it should not be the responsibility of the research community alone to address such questions. Improving understanding of Antarctic decision‐making and situating these understandings within the dynamics of the complex systems that Antarctic governance practices are nested should be among the top priorities of all Antarctic stakeholders, including the decision makers engaged with the governance fora of the ATS.
